# Progressive Vaccinia Acquired through Zoonotic Transmission in a Patient with HIV/AIDS, Colombia

**DOI:** 10.3201/eid2603.191365

**Published:** 2020-03

**Authors:** Katherine Laiton-Donato, Paola Ávila-Robayo, Andrés Páez-Martinez, Paula Benjumea-Nieto, José A. Usme-Ciro, Nicole Pinzón-Nariño, Ivan Giraldo, Diego Torres-Castellanos, Yoshinori Nakazawa, Nishi Patel, Kimberly Wilkins, Yu Li, Whitni Davidson, Jillybeth Burgado, Panayampalli Subbian Satheshkumar, Ashley Styczynski, Matthew R. Mauldin, Martha Gracia-Romero, Brett W. Petersen

**Affiliations:** Instituto Nacional de Salud, Bogotá, Colombia (K. Laiton-Donato, J.A. Usme-Ciro, M. Gracia-Romero);; Hospital Universitario Mayor Méderi-Universidad del Rosario, Bogotá (P. Ávila-Robayo, P. Benjumea-Nieto, N. Pinzón-Nariño, I. Giraldo, D. Torres-Castellanos);; Universidad de La Salle, Bogotá (A. Páez-Martínez);; Universidad Cooperativa de Colombia, Santa Marta, Colombia (J.A. Usme-Ciro);; Centers for Disease Control and Prevention, Atlanta, Georgia, USA (Y. Nakazawa, N. Patel, K. Wilkins, Y. Li, W. Davidson, J. Burgado, P.S. Satheshkumar, A. Styczynski, M.R. Mauldin, B.W. Petersen)

**Keywords:** progressive vaccinia, HIV/AIDS, zoonotic vaccinia, vaccine-preventable diseases, smallpox, Orthopoxvirus, viruses, zoonoses, Colombia, VACV, vaccinia virus

## Abstract

In March 2015, a patient in Colombia with HIV/AIDS was hospitalized for disseminated ulcers after milking cows that had vesicular lesions on their udders. Vaccinia virus was detected, and the case met criteria for progressive vaccinia acquired by zoonotic transmission. Adherence to an optimized antiretroviral regimen resulted in recovery.

Vaccinia virus (VACV) belongs to the genus *Orthopoxvirus* (OPXV) and was the main component of vaccines used during the 1960s and 1970s against smallpox (*1*). More recently, VACV has caused several zoonotic outbreaks in South America (*2*,*3*), where human cases are mainly associated with occupational exposure of farmworkers to infected cows. Progressive vaccinia is a severe and often lethal condition caused by infection and uncontrolled replication of VACV in immunocompromised patients (*4*,*5*).

## The Study

**Figure 1:**
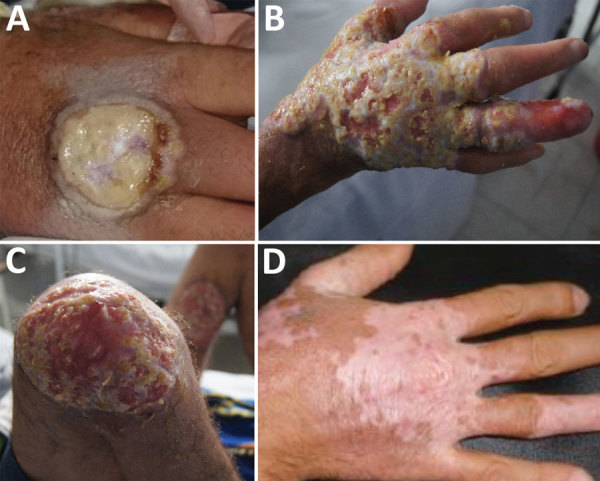
Clinical progression of vaccinia virus infection in a patient with HIV/AIDS, Colombia. A) On December 9, 2014, the patient was referred to the Hospital Universitario Mayor Méderi because of a suppurative ulcer with sharply raised, defined edges on his right hand. B, C) In March 2015, lesions increased in size and disseminated over his face and extremities. D) In July 2015, most lesions completely healed, with mild scarring and depigmentation.

In November 2014, a 30-year-old man with HIV/AIDS living and working at a rural dairy cattle farm in the department of Cundinamarca, Colombia, with no prior hospitalizations for opportunistic infections sought treatment for a suppurative ulcer with initial sharply raised defined edges on his right hand (Figure 1, panel A), right ear, and distal left leg that appeared 1 week after he had milked cows with similar lesions on their udders. He had recently interrupted antiretroviral therapy after onset of depression because of his father’s death 4 months before. Despite treatment with self-formulated antimicrobial drugs and home therapies (application of alcohol, methylene blue, and herbs), the lesions continued and spread within 1 month to his nostrils, glans penis, right leg, right knee, and ankles. On November 14, 2014, the patient was treated with antimicrobial drugs at a local hospital and instructed to comply with his antiretroviral therapy. On December 9, 2014, after failing to respond to treatment, the patient was referred to the Hospital Universitario Mayor Méderi in Bogotá, Colombia. Laboratory tests showed a CD4 cell count of 11 cells/mL and HIV viral load of 44,201 copies/mL. The patient was treated with acyclovir after suspected initial diagnosis of alphaherpesviru*s* infection and was discharged on December 23, 2014, with antimicrobial therapy prophylaxis for opportunistic infections (trimethoprim/sulfamethoxazole) and persuaded to continue his previous antiretroviral therapy (lamivudine/zidovudine and efavirenz).

The patient was readmitted to the hospital on March 24, 2015, because of a deteriorating clinical condition that included deep, severe, and extended foul-smelling ulcers with raised and undefined edges throughout his face and extremities (Figure 1, panels B and C), as well as fever, tachycardia, hearing and vision impairment, anemia, and leukopenia. He received a blood transfusion, prophylactic antimicrobial drugs against opportunistic infections, analgesics, and supportive care. The case was suggestive of poxvirus infection because the patient had not received smallpox vaccination, the pathologic study showed the presence of cytoplasmic B-type inclusion bodies, and the patient reported previous contact with cattle with vesicular lesions in their udders. Therefore, biological samples were remitted to the Instituto Nacional de Salud for viral diagnostics, and, subsequently, to the US Centers for Disease Control and Prevention for confirmation of VACV diagnosis and further characterization.

On March 30, 2015, HIV resistance to antiretroviral drugs was confirmed, and the pharmacologic therapy was changed to raltegravir and darunavir/ritonavir. Within 2 weeks, the lesions had healed considerably, and the patient was discharged from the hospital on April 20. Follow-up visits revealed complete healing of the lesions, mild scarring, and depigmentation (Figure 1, panel D), with the exception of a persistent ulcer on the patient’s left leg. This lesion did not respond to initial antimicrobial treatment or a 2-week course of topical imiquimod.

Experimental assays included ELISA and neutralization tests for OPXV IgM and IgG detection, virus isolation in BSC-40 cells, and molecular detection through OPXV-generic and VACV-specific real-time PCR (Appendix). OPXV IgM and IgG antibodies were detected in serum in March 2015 (5 months after illness onset). IgG but not IgM was in serum in July 2015 (9 months after illness onset). Viral neutralization assays had 50% effective concentration values of 1:517 for the March sample and 1:223 for the July sample (Table). VACV persisted in lesions despite the presence of OPXV IgM and IgG, suggesting that humoral immunity alone might be insufficient to clear infection, as demonstrated previously (*6*). Recovery occurred only after improving adherence and optimizing antiretroviral therapy on the basis of antiretroviral-resistance testing. This finding suggests that cell-mediated immunity is required for complete VACV clearance and that reversing any underlying immunosuppressive condition should be pursued whenever possible for recovery from progressive vaccinia (*7*) (Appendix Figure).

**Table:** Laboratory testing for orthopoxvirus diagnosis in an HIV/AIDS patient who acquired progressive vaccinia through zoonotic transmission, Colombia*

Sample date and type	Serology				PCR		Viral culture
	IgM ELISA	IgG ELISA	Neutralization titer		OPXV-specific (C_t_)	Vaccinia-specific	
2015 Mar							
Serum	Pos	Pos	1:517				
Scab					Pos (31.9)	Pos	Pos
Scab					Pos (28.6)	Pos	Pos
2015 Jul							
Serum	Neg	Pos	1:223				
Scab, left leg					Pos (36.2)	Pos	Pos†
Swab, left leg					Pos (29.2)	Pos	Pos†
2016 Apr							
Paraffin block, left leg					Neg		
Paraffin block, left leg					Neg		
Paraffin block, left leg					Neg		

*C_t_, cycle threshold; Neg, negative; Pos, positive. †Slow-growing.

**Figure 2:**
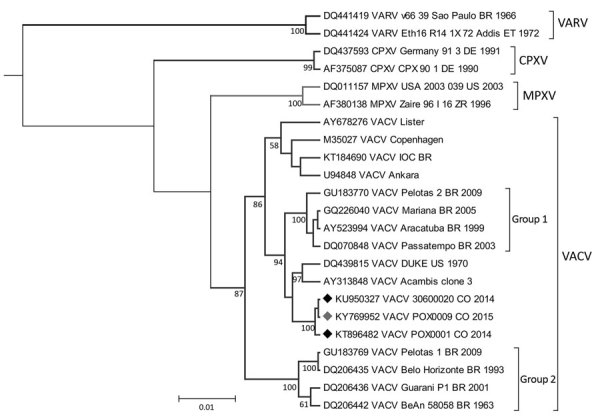
Phylogenetic inference of *Orthopoxvirus* genus based on the A56R hemagglutinin gene. Nucleotide sequences of 829 bp representing the different species were aligned and used for Bayesian inference (Appendix). Black diamonds indicate previously reported sequences of VACV in Colombia; red diamond indicates sequence from the strain from the patient in this study (POX0009). GenBank accession numbers are provided for reference sequences. CPXV, cowpox virus; MPXV, monkeypox virus; VACV, vaccinia virus; VARV, variola virus.

Molecular tests performed on serum and scab samples were positive for OPXV and VACV in March 2015 and remained positive in July 2015 (Table). To better characterize the VACV strain, we used specific primers targeting the A56R hemagglutinin gene (1,134 bp) for amplification and sequencing (*3*). Phylogenetic analysis (Appendix) confirmed infection with a VACV strain whose A56R gene sequence was closely related to those recently reported in Colombia and grouped as a sister lineage of the VACV group 1 in Brazil (Figure 2).

A biopsy of the persistent leg lesion collected in April 2016 tested negative for OPXV DNA by real-time PCR (Table) and positive for *Pseudomonas aeruginosa* and *Escherichia coli* by classic microbiological assays. The lesion healed after focused antimicrobial treatment.

## Conclusions

The clinical case we describe meets all criteria for progressive vaccinia (*4*): immunodeficiency from HIV infection was documented with a CD4 cell count of <50 cells/mL; multiple lesions developed and failed to heal despite antimicrobial therapy; and VACV infection was confirmed by several laboratory methods. Our results document progressive vaccinia acquired through zoonotic transmission.

Because smallpox eradication led to the discontinuation of routine smallpox vaccination before the global spread of HIV, little is known about VACV infections in persons with HIV (*8*,*9*). Progressive vaccinia is thought to occur only in patients with substantial cell-mediated immunodeficiency (*4*). This hypothesis is further supported by the observation that VACV infection (through smallpox vaccination) in 10 HIV-infected persons with CD4 cell counts >200 cells/mL did not lead to progressive vaccinia (*10*). In the case we describe, VACV lesions persisted despite the documentation of VACV neutralizing antibodies. The lesions resolved only after immune reconstitution, indicating that cell-mediated immunity is required for complete viral clearance.

The persistent leg lesion was unexpected given the resolution of the patient’s other lesions and because latent VACV infection has not been described previously. Although testing of this lesion in July 2015 detected VACV, previous studies have demonstrated that VACV can be isolated from smallpox vaccination site lesions even after the separation of scab when the viral infection is presumably recovered (*11*). The positive bacterial cultures and absence of evidence of VACV in the lesion biopsy in April 2016 suggest that this lesion was most likely attributable to secondary bacterial infection resulting from the compromised dermal barrier rather than persistence or reactivation of latent VACV infection.

Our findings suggest that, in VACV infection cases, reversing any underlying immunosuppressive condition should be pursued whenever possible because of the potential role of the cellular immune response in clearing the infection. Because of waning global immunity against OPXVs (*12*), increasing immunosuppressed populations (*13*), and the potential nosocomial (*14*) and demonstrated zoonotic transmission of VACV (*3*), additional infection prevention, treatment, and control strategies are needed.

Appendix Additional information about progressive vaccinia acquired through zoonotic transmission in a patient with HIV/AIDS, Colombia.

## References

[1] Fenner F, Henderson DA, Arita I, Jezek Z, Ladnyi ID. Smallpox and its eradication. 1988 [cited 2019 Sep 19]. https://apps.who.int/iris/handle/10665/39485

[2] Trindade GS, Emerson GL, Carroll DS, Kroon EG, Damon IK. Brazilian vaccinia viruses and their origins. Emerg Infect Dis. 2007;13:965–72. 10.3201/eid1307.061404 18214166 PMC2878226

[3] Usme-Ciro JA, Paredes A, Walteros DM, Tolosa-Pérez EN, Laiton-Donato K, Pinzón MD, et al. Detection and molecular characterization of zoonotic poxviruses circulating in the Amazon region of Colombia, 2014. Emerg Infect Dis. 2017;23:649–53. 10.3201/eid2304.161041 28322708 PMC5367405

[4] Bray M, Wright ME. Progressive vaccinia. Clin Infect Dis. 2003;36:766–74. 10.1086/374244 12627361

[5] Redfield RR, Wright DC, James WD, Jones TS, Brown C, Burke DS. Disseminated vaccinia in a military recruit with human immunodeficiency virus (HIV) disease. N Engl J Med. 1987;316:673–6. 10.1056/NEJM198703123161106 3821799

[6] Bray M. Henry Kempe and the birth of vaccinia immune globulin. Clin Infect Dis. 2004;39:767–9. 10.1086/423005 15472805

[7] Letz AG, McFarland RW, Dice JP. Delay of chemotherapy to prevent progressive vaccinia. Mil Med. 2006;171:788–9. 10.7205/MILMED.171.8.788 16933825

[8] Amorosa VK, Isaacs SN. Separate worlds set to collide: smallpox, vaccinia virus vaccination, and human immunodeficiency virus and acquired immunodeficiency syndrome. Clin Infect Dis. 2003;37:426–32. 10.1086/375823 12884168

[9] Bartlett JG. Smallpox vaccination and patients with human immunodeficiency virus infection or acquired immunodeficiency syndrome. Clin Infect Dis. 2003;36:468–71. 10.1086/368093 12567305

[10] Tasker SA, Schnepf GA, Lim M, Caraviello HE, Armstrong A, Bavaro M, et al.; US Department of Defense Tri-Service AIDS Clinical Consortium. Unintended smallpox vaccination of HIV-1-infected individuals in the United States military. Clin Infect Dis. 2004;38:1320–2. 10.1086/420938 15127348

[11] Pittman PR, Garman PM, Kim SH, Schmader TJ, Nieding WJ, Pike JG, et al. Smallpox vaccine, ACAM2000: Sites and duration of viral shedding and effect of povidone iodine on scarification site shedding and immune response. Vaccine. 2015;33:2990–6. 10.1016/j.vaccine.2015.04.062 25930115

[12] Reynolds MG, Carroll DS, Karem KL. Factors affecting the likelihood of monkeypox’s emergence and spread in the post-smallpox era. Curr Opin Virol. 2012;2:335–43. 10.1016/j.coviro.2012.02.004 22709519 PMC9533834

[13] Parrino J, Graham BS. Smallpox vaccines: Past, present, and future. J Allergy Clin Immunol. 2006;118:1320–6. 10.1016/j.jaci.2006.09.037 17157663 PMC9533821

[14] Zafar A, Swanepoel R, Hewson R, Nizam M, Ahmed A, Husain A, et al. Nosocomial buffalopoxvirus infection, Karachi, Pakistan. Emerg Infect Dis. 2007;13:902–4. 10.3201/eid1306.061068 17553232 PMC2792849

