# Supplementary material for: Progressive Vaccinia Acquired through Zoonotic Transmission in a Patient with HIV/AIDS, Colombia
Source: Emerg Infect Dis. 2020 Mar;26(3):601–5. doi: 10.3201/eid2603.191365 (PMC7045850; doi:10.3201/eid2603.191365)
Supplement: Appendix — Additional information about progressive vaccinia acquired through zoonotic transmission in a patient with HIV/AIDS, Colombia. [file 19-1365-Techapp-s1.pdf]

Article DOI: https://doi.org/10.3201/eid2603.191365

Progressive Vaccinia Acquired through Zoonotic Transmission in a Patient with HIV/AIDS, Colombia

Appendix

Epidemiologic Investigation

The patient's farm is located in the Department of Cundinamarca, Colombia. It comprised ~120 ha and contained >120 cows for milk and meat production; milking was performed manually without the routine use of gloves. Interviews with three farmworkers (excluding the patient described) and one family member did not reveal any active illness or recent history of lesions. Farmworkers reported seeing lesions on the udders and teats of multiple cows (15 out of 35 milking cows) during the time when the patient development lesions. No active lesions were identified during examination of the farm's domestic animals in September 2015.

Serologic Tests

Anti-orthopoxvirus antibodies were determined by enzyme-linked immunosorbent assays (ELISA) from patient (IgM and IgG) and animal (IgG) sera (*1*). Serum samples from the patient were diluted 1:100 for IgG assays and 1:50 for IgM; cutoff values were determined based on the mean plus three standard deviations of negative control serum samples. Additionally, neutralization assay was performed using a Western Reserve Vaccinia virus which expresses the green fluorescent protein (WR-GFP) (*2*); Effective Concentration (EC_{50}) values were calculated for each sample.

Viral Isolation

BSC-40 cells were grown in RPMI medium supplemented with 5% fetal bovine serum and 500 U/mL penicillin; and 50 mg/mL gentamicin. Fresh cultures were inoculated with the biologic sample and the cytopathic effect was observed 6 days after inoculation.

Molecular Characterization

Samples from crusts, swabs, specimens from cutaneous lesions were used for molecular diagnosis and viral culture. DNA was extracted from original samples using DNeasy Blood & Tissue Kit (Qiagen). Forward and reverse primers for detecting poxviruses with low GC content (including Orthopoxvirus) were used for PCR amplification of a short DNA fragment of ≈220 bp (*3*). Additionally, DNA fragments containing the B5R (1130 bp) with the primers designed by Usme-Ciro (Unpublished) Primers B5R_F (5′-ccttttagtgctcgacagtg-3′), B5R_R (5′-atgctctaacggcatcgtag-3′). and A56R genes (Hemagglutinin, 1134 bp) of VACV were amplified (*4*). OPXV-generic qPCR infection was confirmed by OPXV-generic qPCR (E9L non-variola assay) (*5*) and VACV-specific PCR assay from CDC.

Phylogenetic Analysis

DNA sequences from amplicons for A56R gene were obtained using the Applied Biosystems 3130 Genetic Analyzer and the BigDye® Terminator Cycle Sequencing kit v.3.1 (Applied Biosystems, Foster City, CA, USA). Sequences were subsequently assembled through the SeqMan module of the LaserGene® version 7.2.1 (DNASTAR Inc. Madison, WI, USA). Sequences obtained from lesion samples (POX009) were aligned with published A56R gene sequences of VACV using ClustalX2.1 software (*6*). Aligned sequences were used to calculate the best nucleotide substitution model (HKY + gamma) through BIC implemented in jModeltst 2.1.3 software (*7*). Phylogenetic inference was performed with the use of the Bayesian analysis software packages BEAST BEAUti, version 1.8.4 (*8*) and Tracer v1.6 (*9*). The analyses ran a Markov chain Monte Carlo chain length of 10.000.000 and sampling of every 1000 states, with a lognormal relaxed molecular clock (Appendix Figure).

References

1. Karem KL, Reynolds M, Braden Z, Lou G, Bernard N, Patton J, et al. characterization of acute-phase humoral immunity to monkeypox: use of immunoglobulin M enzyme-linked immunosorbent assay for detection of monkeypox infection during the 2003 North American outbreak. Clin Diagn Lab Immunol. 2005;12:867–72. PubMed

2. Johnson MC, Damon IK, Karem KL. A rapid, high-throughput vaccinia virus neutralization assay for testing smallpox vaccine efficacy based on detection of green fluorescent protein. J Virol Methods. 2008;150:14–20. PubMed https://doi.org/10.1016/j.jviromet.2008.02.009

3. Li Y, Meyer H, Zhao H, Damon IK. GC content-based pan-pox universal PCR assays for poxvirus detection. J Clin Microbiol. 2010;48:268–76. PubMed https://doi.org/10.1128/JCM.01697-09

4. Usme-Ciro JA, Paredes A, Walteros DM, Tolosa-Pérez EN, Laiton-Donato K, Pinzón MD, et al. Detection and molecular characterization of zoonotic poxviruses circulating in the Amazon region of Colombia, 2014. Emerg Infect Dis. 2017;23:649–53. PubMed https://doi.org/10.3201/eid2304.161041

5. Li Y, Olson VA, Laue T, Laker MT, Damon IK. Detection of monkeypox virus with real-time PCR assays. J Clin Virol. 2006;36:194–203. PubMed https://doi.org/10.1016/j.jcv.2006.03.012

6. Larkin MA, Blackshields G, Brown NP, Chenna R, McGettigan PA, McWilliam H, et al. Clustal W and Clustal X version 2.0. Bioinformatics. 2007;23:2947–8. PubMed https://doi.org/10.1093/bioinformatics/btm404

7. Darriba D, Taboada GL, Doallo R, Posada D. jModelTest 2: more models, new heuristics and parallel computing. Nat Methods. 2012;9:772. PubMed https://doi.org/10.1038/nmeth.2109

8. Bouckaert R, Vaughan TG, Barido-Sottani J, Duchêne S, Fourment M, Gavryushkina A, et al. BEAST 2.5: an advanced software platform for Bayesian evolutionary analysis. PLOS Comput Biol. 2019;15:e1006650. PubMed https://doi.org/10.1371/journal.pcbi.1006650

9. Rambaut A, Drummond AJ, Xie D, Baele G, Suchard MA. Posterior summarisation in Bayesian phylogenetics using Tracer 1.7. Syst Biol. 2018;67:901–4. PubMed https://doi.org/10.1093/sysbio/syy032

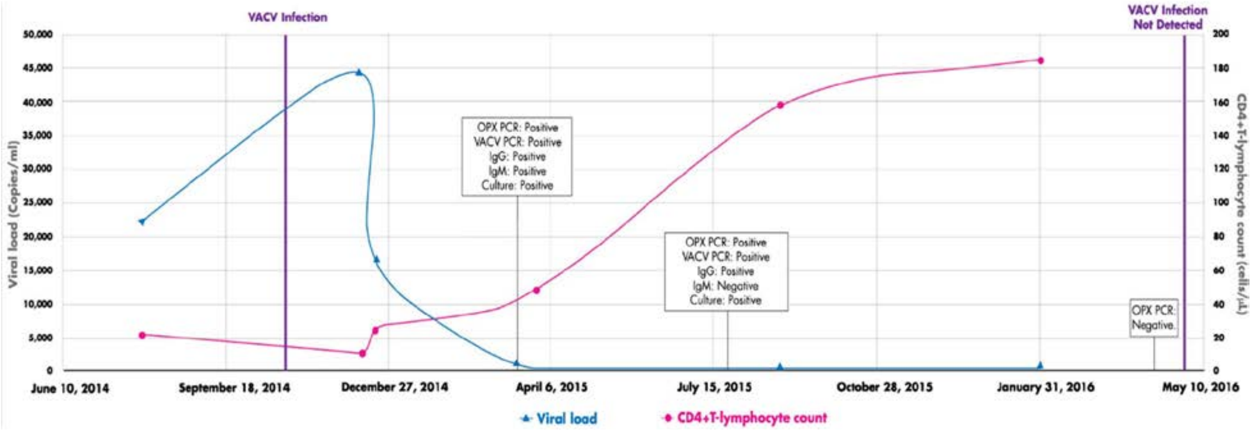

Appendix Figure. HIV viral load and absolute CD4+ count during VACV infection. Changes in HIV load (blue line) and absolute CD4+ counts (pink line) between July 2014 and February 2016. Purple lines indicate the duration of VACV infection. Gray lines indicate sample collection for OPXV diagnosis. OPX PCR: Orthopoxvirus PCR test; IgG: Anti-orthopox IgG antibodies; IgM: Anti-orthopox IgM antibodies; Culture: Viral culture in green monkey kidney cells (BSC40).
